# The decision‐making process and criteria in selecting candidate drugs for progeria clinical trials

**DOI:** 10.15252/emmm.201606280

**Published:** 2016-05-27

**Authors:** Leslie B Gordon, Mark W Kieran, Monica E Kleinman, Tom Misteli

**Affiliations:** ^1^ Department of Anesthesiology, Perioperative and Pain MedicineBoston Children's Hospital and Harvard Medical SchoolBostonMAUSA; ^2^ Department of PediatricsHasbro Children's Hospital and Warren Alpert Medical School of Brown UniversityProvidenceRIUSA; ^3^ Dana‐Farber Boston Children's Cancer and Blood Disorder CenterDana‐Farber Cancer InstituteBostonMAUSA; ^4^ National Cancer InstituteNIHBethesdaMDUSA

**Keywords:** Genetics, Gene Therapy & Genetic Disease, Pharmacology & Drug Discovery

## Abstract

Hutchinson–Gilford progeria syndrome (progeria) is an extremely rare premature aging disease with a population prevalence of 1 in 20 million. Nevertheless, propelled by the discovery of a causal mutation in the lamin A/C gene (*LMNA*) (De Sandre‐Giovannoli *et al*, 2003; Eriksson *et al*, 2003) and strong patient advocacy (Gordon & Gordon, 2014), progeria has rapidly become a vibrant field of study, attracting a wide range of researchers from basic cell biologists to clinicians.

Progeria has catalyzed the field of lamin biology and has created a new avenue for aging research, particularly because progerin, the disease‐causing aberrant lamin A protein, is not only generated in affected children, but is also generated at comparatively lower levels in the vasculature and other tissues of non‐progeria individuals (Gordon *et al*, 2014b). Peer‐reviewed publications on progeria have increased by an order of magnitude from an average of < 6 per year from 1921 to 2002 to 67 per year since its gene discovery in 2003.

Much effort in the field is now dedicated to finding drugs for therapeutic application that could save children with progeria, who die primarily from heart attacks due to accelerated atherosclerosis at an average age of 14.7 years (Gordon *et al*, 2014a). In just over a decade, a remarkable number of potential therapeutic candidate molecules for progeria have been identified, many in basic research laboratories. This includes the first‐ever treatment, lonafarnib, which was derived from a mechanistic understanding of the posttranslational processing of progerin. It emerged with extraordinary speed and a clinical trial was conducted < 5 years after the gene discovery (Gordon *et al*, 2012). Related trials (https://clinicaltrials.gov/ct2/results?term=progeria&cond=%22Progeria%22) followed in quick succession.

As we emerge from the early post‐mutation discovery era in progeria, a considerable number of drug candidates have been identified either through focused, pathway‐based investigations or through unbiased high‐throughput screening. This represents a tremendous advance toward new treatments and cure, but also poses challenges that are shared with most other rare diseases. Two major elements drive these challenges: an exceedingly small number of perspective trial participants, and limited natural history data that can be used to develop short‐term, clinically meaningful treatment efficacy readouts. Whereas common disease groups can conduct many large‐cohort natural history and treatment trials simultaneously, most rare disease groups cannot. For example, currently only about 100 children living with progeria are identified worldwide, and progeria clinical trials require a minimum of 2 years of treatment for efficacy readout. Consequently, it is not possible to conduct more than a few small clinical trials at once. Treatments may need to be prioritized based on the answers to key questions about how candidates are selected for clinical testing, such as: What are the preclinical data and safety standards for a candidate drug to advance into a clinical trial? How do the scientific, medical, and research advocacy communities work together to ensure that candidates with the greatest chances for success in saving children with progeria are prioritized, and evaluated with the greatest possible speed for this fatal disease?

## Candidate drug discovery

Many drug candidates are initially identified in cell‐based assays using reversal of typical cellular disease defects as readouts. In the case of progeria, there are a number of valid *in vitro* readouts of cellular improvement. The standard first‐line assay is the normalization of nuclear shape, which is severely deformed in patient cells, likely by the presence of progerin embedded within the nuclear membrane (Eriksson *et al*, 2003; Gordon *et al*, 2014b). In addition, evidence for mechanism of treatment effect, cellular toxicity studies, and effect on progerin levels and progerin localization are highly supportive factors for discovering drug candidates.

Murine models of progeria have advanced significantly in the last 5–7 years, with the generation of several progerin‐producing models that develop vascular disease and have some overlap with human disease phenotypes (Zhang *et al*, 2013). Although other progeroid mouse model testing can contribute to our understanding of disease, these particular models should now serve as the principle *in vivo* murine testing systems for candidate drugs. Toxicity testing in progeria mice is appropriate even if a drug is well characterized in non‐progeria populations. While the crossover to human success is low (Herter‐Sprie *et al*, 2013), efficacy testing in animal models is still desirable whenever possible. As a general rule, the less clinical experience there is with a candidate drug, the more animal model efficacy support will be required.

## Moving candidate drugs into consideration for human trials

In progeria, as in other rare diseases, emerging candidate drugs can be divided loosely into three categories based on the degree of clinical experience in non‐progeria populations. The requirements for cellular and/or animal model support will depend on the strength of many factors, both between and within the groups: (i) Commercially available or investigational compounds with acceptable tolerability (e.g. everolimus, sulforaphane, resveratrol, methylene blue. Advancement to trial status will depend on volume and characteristics of clinical experience, whether there is pediatrics experience, profiles for toxicity, tissue delivery, pharmacokinetics, pharmacodynamics, and efficacy. For example, identifying a well‐established, low‐toxicity, pediatric drug makes a rapid translation to the clinic easier, whereas an investigational drug with less desirable toxicity will warrant rigorous progeria mouse testing prior to implementation. (ii) Clinically acceptable analogues of compounds that have shown potential benefit when tested in cultures or mouse models (e.g. rapamycin, retinoic acid), where the tested compounds are unsuitable for use in progeria clinical treatment. Examples of impediments to implementation for these compounds include requirement for toxicity monitoring through hematologic testing that surpasses the safe maximum blood volume that these small children can provide, essential toxicity monitoring that will not withstand the worldwide patient locations, or serious known side effects that prohibit long‐term use. (iii) Compounds with no clinical experience/experimental compounds in development (e.g. NAT10, ICMT inhibitors, RNA and gene editing therapies). This is the largest group by far, and since these compounds have no accompanying clinical experience, they will require rigorous preclinical toxicity testing, and most likely some evidence of efficacy in a progeria animal model.

When optimizing the design of studies aimed at supporting clinical trials, basic scientists should consider important details that can have a significant impact. First, testing the drug of interest for the trial itself, and not merely an analog or drug in the same family, is ideal. This is mainly because drug toxicity is often due to off‐target effects inherent to the specific drug formulation. For example, lonafarnib has a much different toxicity profile from its analog tipifarnib. Second, including a study arm that tests a drug combination that might be considered in a trial (e.g. lonafarnib plus new compound of interest) can save both time and expense over conducting an entirely new study once supporting data for a new drug of interest are identified.

## Optimizing measures of treatment outcome is pivotal for rare diseases

A sizeable obstacle to carrying out multiple drug trials in a rare disease is the severely limited number of available patients. Hence, developing concrete, morbidity‐relevant, objectively measurable primary outcome measures is paramount to successful assessment of whether a drug has influenced disease. Better detection of treatment effect can facilitate smaller patient cohorts per trial arm and increase the likelihood that more than one trial, or multiple trial arms, can be achieved if needed. Although the rate of weight gain has been utilized in progeria trials because it is reliably trackable and abnormal in all patients, cardiovascular measures would be preferred. Through natural history studies, vascular echodensity and pulse wave velocity have appeared as viable outcome options (Gordon *et al*, 2012), but additional rigorous natural history studies are still needed in order to further estimate untreated disease trends for these and other cardiovascular outcomes. These efforts will improve the detection of treatment effect on the smallest possible cohort size.

New approaches to clinical trial design do not obviate the primary function of performing these trials, which is to identify an agent(s) that can impact the clinical disease in a meaningful way and with acceptable toxicity. Adaptive designs, where multiple different therapies are tested, with accrual weighted to those arms showing the most promise, can help eliminate inactive therapies more rapidly while focusing limited patients into trial arms with the most promise (http://www.ema.europa.eu/docs/en_GB/document_library/Scientific_guideline/2009/09/WC500003615.pdf). All of these, however, require the ability to detect success in the shortest time span possible and with the smallest number of patients. Identifying specific measures of clinical improvement or validated biomarkers continues to be a major impediment to the rapid identification of active drugs in progeria. This highlights the importance of optimizing our understanding of the biology of the disease, so that actionable endpoints can be identified.

## Success takes communication

Coordination of efforts across the basic‐to‐clinical spectrum is critical to timely progress in bringing new drugs for rare diseases to the clinic. It is key that frequent and open communication occurs among members of the often small, but global, community. This can be done through scientific meetings where unpublished information is presented, and through community‐based means to make data available prior to publication, such as pre‐print servers. Importantly, unpublished data are often useful well prior to publication to inform and stimulate additional research and preparations needed for trial implementation. For example, *in vitro* data on a compound may simultaneously trigger murine studies, generate interest from pharmaceutical companies, and initiate clinical trial strategic planning discussion, without jeopardizing peer‐review publication of the source data. In addition, early input from appropriately experienced clinicians can assure that preclinical experiments are designed by basic scientists with the foresight needed to meet the approval standards required by clinical trial review bodies (e.g. U.S. FDA or equivalent, academic institutions, funding organizations, and drug companies). This “layered” approach is pivotal in accelerating the path from basic discovery to the clinic.

In addition, there is tremendous value in research advocacy organizations that can often connect basic scientists with clinical researchers who can advise on which drugs to use in preclinical studies, recruit pharmaceutical companies to help bring chemicals of interest through the rigors of testing and on to clinical studies, plan for trial funding, and facilitate patient recruiting by connecting potential trial participants with trial investigators. The global nature of many rare disease trials, including those for progeria, means that the clinical trial team must participate in clear discussions with patients' families and their local physicians in their native languages regarding support for, and potential risks of planned trials. This multistage process takes early and frequent communication.

## Forward progress is complicated but critical

In sum, decisions about moving ahead with a clinical trial for children with progeria, or any rare disease, are complex and are never based on a single set of criteria. These decisions must be evaluated with a holistic and situational view. In the face of the inherent challenges around conducting clinical trials on extremely rare diseases, as described here for progeria, there is a reality that lack of treatment has a known and devastating outcome. Preclinical studies give us invaluable leads about what might be effective in patients, but ultimately only human trials can tell us about safety and efficacy. Therefore, while pursuing new treatment avenues, any treatment that meets acceptable standards to advance to trial should be vigorously, but diligently, pursued.

## Conflict of interest

LBG is the volunteer Medical Director for The Progeria Research Foundation. TM and MEK are members of The Progeria Research Foundation volunteer Medical Research Committee.
